# Comparison of normal values of Duplex indices of vertebral arteries in young and elderly adults

**DOI:** 10.1186/1476-7120-7-2

**Published:** 2009-01-13

**Authors:** Masoud Nemati, Abolhassan Shakeri Bavil, Naser Taheri

**Affiliations:** 1 Department of Radiology, Tabriz University of Medical Sciences, Tabriz, Iran

## Abstract

**Background:**

Considering the role of aging in brain atrophy and cerebral vascular demand, we carried out this study to clarify the role of aging in duplex indices of vertebral arteries.

**Methods:**

From June 2005 to June 2006, 96 volunteers with age range of 20 to 95 years, were evaluated with color doppler for duplex indices of vertebral arteries. Sever hemodynamic stenosis was excluded in all of these patients. These volunteers were subdivided in two groups: younger and older than 60 year old. In all of these patients we measured diameter, peak systolic velocity (PSV), resistive index (RI), and flow volume (FV) of vertebral arteries in right and left sides.

**Results:**

There was no significant difference in diameter, PSV, RI and FV between two groups. We have clarified that in patients younger than 60 year old, comparing right and left vertebral arteries, PSV and FV were higher in left side.

**Conclusion:**

Duplex indices of vertebral arteries are age independent in adults.

## Background

The vascular system of human brain differs from other organs in the body, although it accounts for only 2% of the body weight, the brain receives 15% of the body oxygen supply in the basal state [1].

The brain is supplied directly by four vessels: the two internal carotid and two vertebral arteries.

Doppler ultrasound is an established technique for evaluating extracranial portion of carotids and vertebral arteries in patients with manifested or suspected cerebrovascular diseases, not only for screening but also for preoperative assessment [2].

With increasing age, due to brain atrophy, oxygen demand and total cerebral blood Flow decrease [3].

Corttical and subcortical atrophy in the elderly may be more relevant for carotid flow volume than for the vertebral system because the brain stem is not so much affected by the aging process [4].

Muller and coworkers [5] and also Martin [6] have been shown a significant decrease in basilar and vertebral arteries flow velocities with increasing of age, but in someother studies there was not any significant difference in diameters, velocities and flow volumes of vertebral arteries with aging [4,7]. Considering these contraversial findings, we decided to compare blood flow and duplex indices of vertebral arteries in volunteers younger and older than 60 year old to clarify the effect of aging in vertebrobasilar circulation.

## Methods

In this prospective study, from June 2005 to June 2006, using a 7.5 MHZ linear transducer of a Hitachi EUB 525 system, we examined vertebral arteries in 96 volunteers. The youngest patient was 23 year old and the oldest one was 95 year old (age range: 23–95 years). These patients were subdivided in two groups: in groups A, 40 patients younger than 60 year old were studied (age range: 23–57 years, mean age: 36.25 years) including 26 males and 14 females, in group B, 56 patients older than 60 years were examined (age range: 60–95 years, mean age: 69.21 years) including 32 males and 24 females.

We examined right and left vertebral arteries in all of volunteers and therefore 192 arteries were examined (80 in group A, 96 in group B). In all of these vessels cross sectional mean diameter, resistive index (RI), peak systolic velocity (PSV) and flow volume (FV) were measured at second part of vertebral artery (at C4 – C5 level). The angle of insonation in all of our patients was under 60 degree, to reduce the measurement bias, and all of measurements were repeated two or three times and all of declared figures were the average of these measurements.

In all of patients in both groups, sever hemodynamic stenosis of carotid arteries was excluded by doppler examination of common and internal carotid arteries. Finally using Independent T test, all findings were analyzed.

### Ethics

An ethical approval to this resaerch was obtained from Research Vice Chancellor office of Tabriz University of Medical Sciences, Tabriz, Iran.

In addition, a verbal informed consent reagrding the use of patients' details in publication was obtained from the entire subjects.

## Results

Mean diameter of vertebral arteries was 3.25 mm with standard deviation of 0.55 in group A and 3.42 mm with standard deviation of 0.62 in group B, and there was not any significant difference between two groups (p = 0.292) and also there was not any meaningful difference between right and left vertebral arteries (p = 0.778).

Mean value for RI was 0.69 (SD = 0.06) in group A and 0.71 (SD = 0.06) in group B (p = 0.595).

PSV by centimeter per second in group A and B was prospectively 39.92 ± 14.54 and 36.44 ± 12.93 and in comparing two groups, p was 0.949.

In group B there was not any significant difference in PSV between right and left sides (p > 0.05) but in group A (younger age group) PSV was significantly higher in left vertebral artery than right side (p = 0.02).

Flow volume (FV) was calculated based on time averaged maximum velocity by following equation:

FV = (Time Averaged maximum velocity/2) × Area.

Average FV was 119.15 ml/min with standard deviation of 46.04 in group A and 117.89 ml/min with standard deviation of 50.99 in group B and the difference was not significant (p = 0.76).

**Figure 1:**
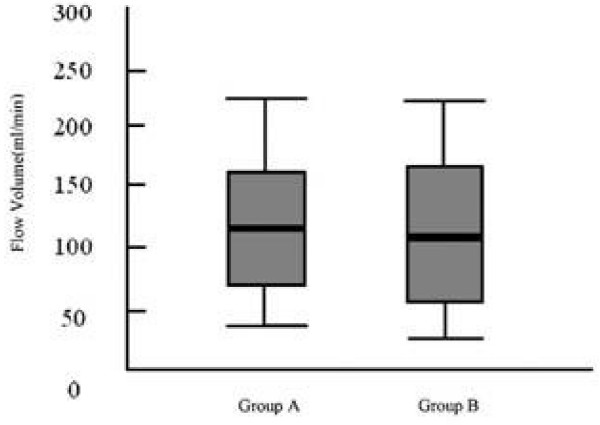
**Mean vertebral artery flow volumes for group A (younger than 60 years) and group B (older than 60 years)**.

**Figure 2:**
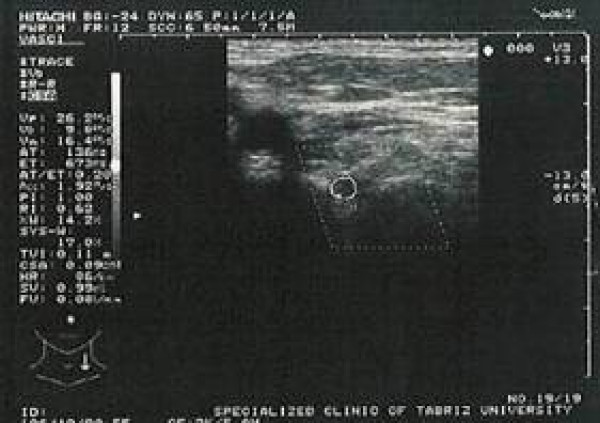
**Typical Duplex tracing of a vertebral artery**.

In group A mean value of FV in right side was 101.01 and in left side it was 137.29 and the difference between right and left was statistically significant (p = 0.02) and interindividual variability was not so high and when we recalculated FV in right and left side by 25% and 75% quartiles the p-value as before was meaningful (p = 0.04) but in group B this difference was not significant (109.53 versus 126.25 and p = 0.8) (see figures 1 and 2)(Table 1).

**Table 1:** Doppler values of vertebral arteries in different age group

	20–40 y	40–60 y	60–80 y	>80 y
Diameter (mm) mean value ± SD	3.51 ± 0.53	3.33 ± 0.55	3.39 ± 0.64	3.37 ± 0.61

RI mean value ± SD	0.701 ± 0.06	0.67 ± 0.05	0.70 ± 0.07	0.73 ± 0.06

PSV (cm/s) mean value ± SD	41.81 ± 14.68	37.61 ± 14.59	37.65 ± 12.7	38.90 ± 13.1

FV (ml/min) mean value ± SD	130.03 ± 47.09	110.52 ± 46.01	122.19 ± 51.02	112.19 ± 52.71

## Discussion

With increasing age because of brain atrophy, oxygen demand and total cerebral blood Flow decreases but our study shows that this decrease is not significant in vertebrobasilar system and in vertebral arteries; diameter, RI, PSV and FV don't change significantly with aging. These findings maybe because of lesser degree of atrophy in brainstem comparing with cerebral hemispheres in elders.

Our findings are discordant with results of Muller [5] and Martin [6] but concordant with Seidel and coworker's study [3] which has highlighted age independence of doppler indices of vertebral arteries.

An interesting finding in our study is difference between right and left vertebral arteries in patients younger than 60 year old. We have showed that in young adults peak systolic velocity (PSV) and flow volume (FV) in left vertebral artery are significantly higher than right side. We think that this is because of anatomical difference of right and left vertebral arteries; in right side, subclavian artery originates from brachiocephalic artery but left subclavian artery directly originates from aorta, beside this branching angle of left vertebral artery from subclavian and also branching angle of left subclavian from aorta is more acute than same angles in right side.

## Competing interests

The authors declare that they have no competing interests.

## Authors' contributions

MN contributed to Duplex ultrasound studies and also wrote the manuscript. ASB conducted Duplex ultrasounds. NT conducted the statistical analysis and contribued to the writing of the manuscript.
